# Faecal microbiome and metabolic signatures in rectal neuroendocrine tumors

**DOI:** 10.7150/thno.66464

**Published:** 2022-01-31

**Authors:** Wei Hu, Ze Min Chen, Xia Xi Li, Lan Lu, Gen Hua Yang, Zheng Xia Lei, Li Juan You, Xiao Bing Cui, Si Cun Lu, Zhi Yong Zhai, Zhi Yu Zeng, Ye Chen, Si Lin Huang, Wei Gong

**Affiliations:** 1 Department of Gastroenterology, Shenzhen Hospital, Southern Medical University, Shenzhen, Guangdong, China; 2 The Third School of Clinical Medicine, Southern Medical University, Shenzhen, Guangdong, China; 3 Antibiotics Research and Re-evaluation Key Laboratory of Sichuan Province, Sichuan Industrial Institute of Antibiotics, School of Pharmacy, Chengdu University, Chengdu, Sichuan, China; 4 Department of Gastroenterology, South China Hospital, Health Science Center, Shenzhen University, Shenzhen, Guangdong, China

**Keywords:** Rectal neuroendocrine tumor, Gut microbiota, Metabolite, Shotgun metagenomic sequencing, Untargeted liquid chromatography-mass spectrometry (LC-MS) metabolomics.

## Abstract

**Background:** The prevalence of rectal neuroendocrine tumors (RNET) has increased substantially over the past decades. Little is known on mechanistic alteration in the pathogenesis of such disease. We postulate that perturbations of human gut microbiome-metabolome interface influentially affect the development of RNET. The study aims to characterize the composition and function of faecal microbiome and metabolites in RNET individuals.

**Methods:** We performed deep shotgun metagenomic sequencing and untargeted liquid chromatography-mass spectrometry (LC-MS) metabolomic profiling of faecal samples from the discovery cohort (18 RNET patients, 40 controls), and validated the microbiome and metabolite-based classifiers in an independent cohort (15 RNET participants, 19 controls).

**Results:** We uncovered a dysbiotic gut ecological microenvironment in RNET patients, characterized by aberrant depletion and attenuated connection of microbial species, and abnormally aggregated lipids and lipid-like molecules. Functional characterization based on our *in-house* and Human Project Unified Metabolic Analysis Network 2 (HUMAnN2) pipelines further indicated a nutrient deficient gut microenvironment in RNET individuals, evidenced by diminished activities such as energy metabolism, vitamin biosynthesis and transportation. By integrating these data, we revealed 291 robust associations between representative differentially abundant taxonomic species and metabolites, indicating a tight interaction of gut microbiome with metabolites in RNET pathogenesis. Finally, we identified a cluster of gut microbiome and metabolite-based signatures, and replicated them in an independent cohort, showing accurate prediction of such neoplasm from healthy people.

**Conclusions:** Our current study is the first to comprehensively characterize the perturbed interface of gut microbiome and metabolites in RNET patients, which may provide promising targets for microbiome-based diagnostics and therapies for this disorder.

## Introduction

Gastroenteropancreatic neuroendocrine neoplasms (GEP-MENs) refer to a relatively rare but clinically important aggregation of malignancies, accounting for ≤ 1% of all neoplasms 1-3. As the second most common digestive cancer in terms of prevalence, GEP-MENs are defined as deprivation of normal epithelial tubular gland architectures and the expression of neuroendocrine markers such as chromogranin A (CgA) or synaptophysin (SYN) 4. WHO classification system, mainly based on the proliferative index, subdivided these malignancies into well differentiated MENs (Neuroendocrine tumors, NETs; grade (G) 1, Ki-67 index ≤ 2%; or G2, Ki-67 index 3 - 20%), and poorly differentiated MENs (Neuroendocrine carcinomas, NECs; G3, Ki-67 index ≥ 20%) 3-5. Most recent evidence confirmed repeatedly that the annual incidence rate of GEP-MENs was continuously increased over the past three decades 1, probably due to the widespread use of screening colonoscopy, presenting great challenges for clinical management.

Rectum is the second most primary MEN site in the gastrointestinal tract, only next to small intestine (30.8%), accounting for 26.3% of all GEP-MENs 3, 6-8. Unlike midgut MENs mostly diagnosed in white individuals, higher frequency of rectal MENs tends to develop in Asian, African American, and Native American patients. According to the Surveillance, Epidemiology, and End Results (SEER) registry database, the incidence of rectal MENs has increased by 800 - 1000% in the last 35 years in US 9. Similar findings were reported in Far East, including China, Japan, and Taiwan 10-12. In contrast with other GEP-MENs, most (> 90%) rectal MENs were detected as small and single polyps (< 1 cm), with well-differentiated histological morphology (G1, G2). Rectal MENs are usually asymptomatic and featured by slow proliferation. As such, treatment delays often occur, resulting in incurable once neoplastic cells metastasize to distant locations 8. Currently, surgical resection is still the standard treatment for rectal MENs patients without distant metastases, whereas options for unresectable patients are limited. Further understanding of the aetiology and pathogenesis of rectal MENs will provide deep insight for novel diagnostic and therapeutic advances for patients with this disease.

A large body of evidence has highlighted the decisive role of gut microbiota in establishment and maintenance of human health 13. This community exerts broad modulation effects on host, including nutrient synthesis, metabolizing indigestible carbohydrates, preventing the colonization of pathogenic microbes, promoting maturation of host immune system, and maintenance of colon mucosal integrity 14. Gut microbiome affects the host not only through the destructive effect of certain pathogens, but also by means of metabolites, which are small molecules derived from bacterial metabolism of dietary substrates, or directly produced by gut microbes. Disordered intestinal flora and metabolite profiles have been documented, also implicated in the pathogenesis of diverse gastrointestinal disorders 15. For example, Franzosa *et al*. uncovered a strong correlation of specific classes of metabolites such as short chain fatty acids (SCFA) with intestinal inflammation and IBD 16. SCFA, mainly consist of acetate, propionate and butyrate, are the end-products produced by gut microbiota through saccharolytic fermentation of non-digestible carbohydrates in small and large intestines. Specific bacterial species responsible for SCFA production have been well characterized by genomic sequencing approaches. Butyrate synthesis was predominantly contributed by *Ruminococcus bromii* via fermentation of resistant starch in human colon 17, while mucin was fermented into propionate mostly by *Akkermansia municiphilla* 18. These studies collectively highlighted that the interdependence between gut microbiota and metabolites are closely associated with profound effects on host health.

Dysbiosis of gut microbiome and metabolites has been widely studied in various types of digestive tract diseases, however, to date, partly due to the relatively low number of patients, we still know little about how specific bacterium and metabolites shape the colorectal environment, thus, to cause, sustain, mitigate, or predict the rectal diseases such as rectal neuroendocrine tumors (RNET). To address whether gut microbiota and metabolites contribute to the pathogenesis of RNET, our current study performed deep shotgun metagenomics and untargeted liquid chromatography-mass spectrometry (LC-MS) metabolomic profiling on 92 stool samples from two cohort, the 58-case discovery cohort (40 healthy controls, 18 RNET patients) and the 34-case validation cohort (19 healthy controls, 15 RNET patients), aiming at characterizing the composition and function of faecal microbial community and metabolites from patients diagnosed as RNET. With these unbiased approaches, for the first time, we presented comprehensive characterization regarding specific microbiome and metabolites in RNET patients, with the intention of yielding further insights into the details of RNET pathogenesis and the implication of disease-related microbial dysbiosis.

## Materials and Methods

See details in the supplementary experimental procedures.

## Results

In total, 58 eligible cases in the discovery cohort including 18 treatment-naïve patients diagnosed as RNET and 40 healthy individuals were included in this study. Clinical characteristics for each subject were provided in Table S1 and Figure S1A. Smoking and alcohol consumption history differed between groups without significance (Figure S1B, Brinkman index, *p* = 0.75; Alcohol, *p* = 0.089, two-tailed wilcoxon rank-sum test). RNET patients in our cohort tended to have higher age and BMI index, although we didn't find statistical significance regarding these clinical parameters between RNET and healthy individuals (Figure S1B, Age, *p* = 0.12; BMI, *p* = 0.36; two-tailed wilcoxon rank-sum test).

**Figure 1:**
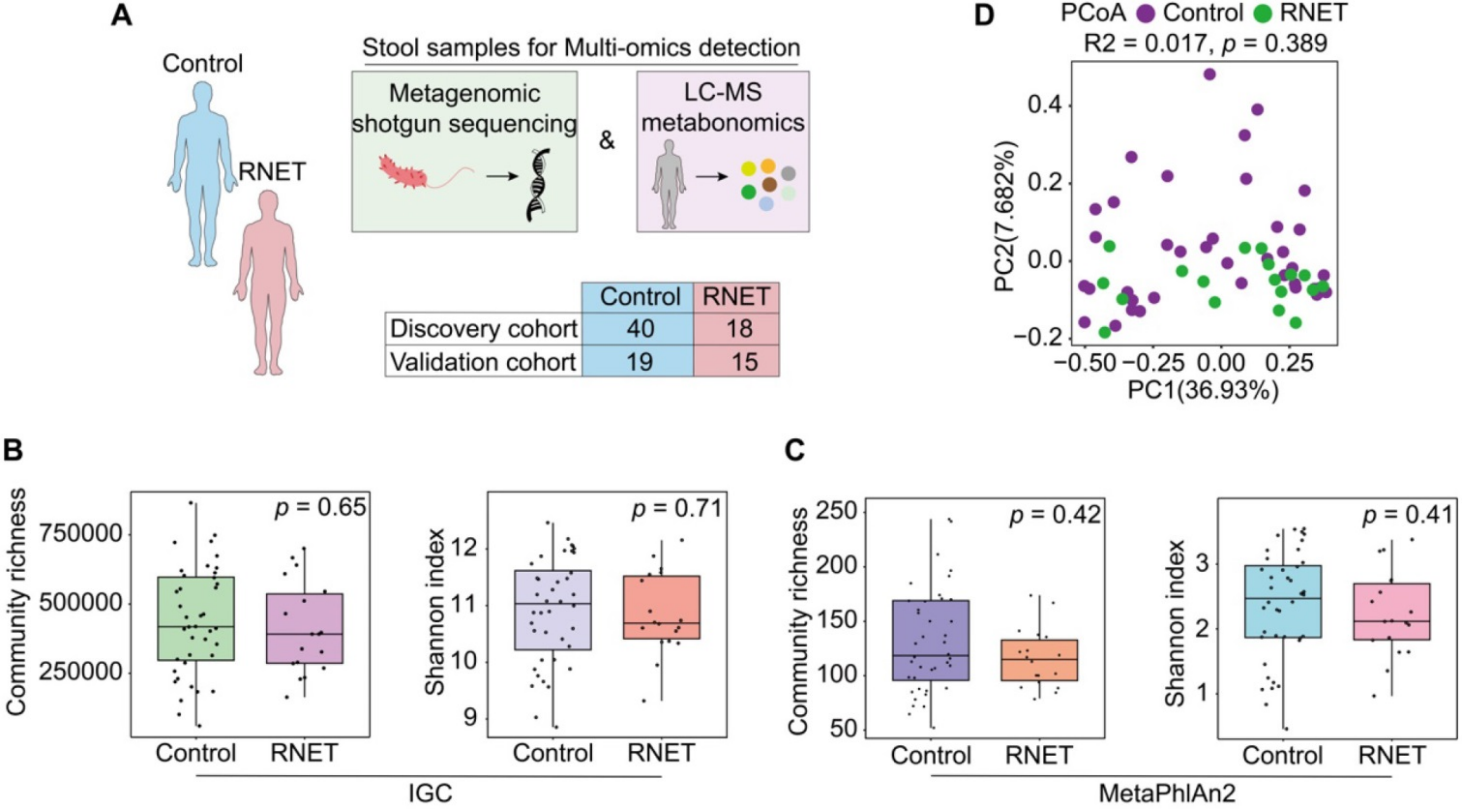
** Faecal microbiome structure in RNET and control individuals.** (A) We collected 92 stool samples from two cohort, the 58-case discovery cohort (40 healthy controls, 18 RNET patients) and the 34-case validation cohort (19 healthy controls, 15 RNET patients). Each faecal sample was subjected for deep shotgun metagenomic sequencing and LC-MS metabonomic profiling. (B-C) Microbiome community richness and Shannon index (α-diversity) at gene and species levels were measured using IGC (B) and MetaPhlAn2 (C) annotation, respectively. The dot represents one value from individual participants. Lines in the boxes indicate medians, the width of the notches is the IQR, the lowest and highest values within 1.5 times the IQR from the first and third quartiles. *p* values were calculated by two-sided Wilcoxon rank sum test. (D) Principal coordinates analysis (PCoA) with Bray-Curtis distance (β-diversity) of the discovery cohort based on gut metagenomic species profiles. *p* values were calculated by two-sided Wilcoxon rank sum test. ADONIS, R^2^ = 0.017, *p* = 0.389.

To comprehensively characterize the gut microbiome and metabolic features in RNET patients, we carried out highthroughput shotgun metagenomic sequencing on 58 faecal samples to profile their microbial community taxonomic composition. Then, each sample was further explored by LC-MS metabolomics in non-targeted modes, in order to capture a mass of established and uncharacterized metabolites (Figure 1A). Metagenomic and metabolomic data were analyzed for: 1) disease specific alteration in individual's microbial and metabolic features; 2) association between disease abundant microbiota and metabolites; 3) potential value of these features in RNET diagnosis. Specific features discovered in this cohort were then validated against an independent cohort of 15 RNET patients without receiving preoperative chemoradiotherapy and 19 healthy controls.

### RNET-specific microbial community structure identified by shotgun metagenomic sequencing

Metagenomic sequencing was performed and generated an average of 71.36 million clean reads (11 Gb of data) per faecal sample on an NovaSeq 6000 platform. High-quality reads were then aligned to the updated gut microbiome gene catalog, representing the microbiome of the discovery cohort. To assess the difference of bacterial diversity between groups, we aligned sequences for gene count and α-diversity. The Integrated Gene Catalog (IGC) (Figure 1B) and Metagenomic phylogenetic analysis (MetaPhlAn2) (Figure 1C) results didn't show statistical significance between groups, although consistent reduction of community richness and Shannon index were observed in RNET patients compared with their control counterparts (IGC: Community richness, *p* = 0.65, Shannon index, *p* = 0.71; MetaPhlAn2: Community richness, *p* = 0.42, Shannon index, *p* = 0.41; two-tailed wilcoxon rank-sum test), which is indicative of delicate alterations of gut bacterial composition in RNET individuals. Next, by performing analysis including Principal Component Analysis (PCA), Principal Coordinates analysis (PCoA) and Nonmetric MultiDimensional Scaling (NMDS), we evaluated the overall gut microbial structure in RNET patients at the phylum, genus, and species levels, respectively. Similarly, we didn't find distinct separation of microbiome composition at these levels (Figure S2, Table S2). Analysis of similarities (ANOSIM) with Bray-Curtis distance also showed marginal difference between these two groups at the three dimensions (Figure S2). Ordination of Bray-Curtis dissimilarity by PCoA analysis at the species level was presented in Figure 1D (Adonis: R^2^ = 0.02, *p* = 0.389). As BMI, smoking and alcohol history have been implicated as potential risk factors for MENs 19, we next adjusted bacterial diversity for these parameters using multivariate association with linear models (MaAsLin2) method (Table S3-5). Here we found that bacterial α and β-diversity were not significantly influenced by gender, age, BMI, smoking and alcohol history. Thus, our findings collectively indicate mild shift of gut microbiome structure in patients with RNET.

### Species-level changes in RNET microbiome community composition

**Figure 2:**
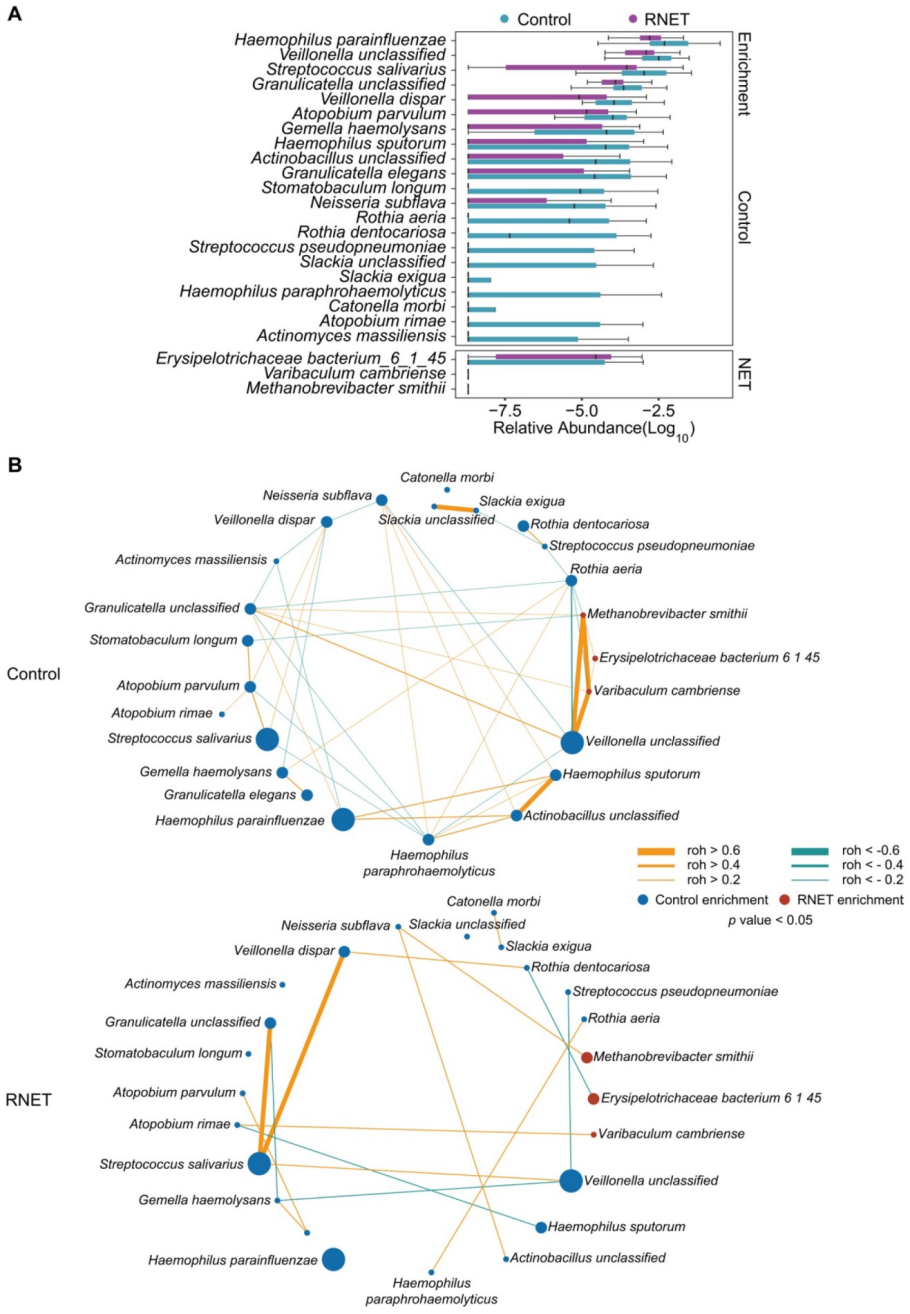
** Species-level changes in RNET microbiome community composition.** (A) Relative abundance of microbial species showed significant difference between RNET patients and controls (*p* < 0.05, two-sided Wilcoxon rank-sum test). Boxes represent the IQRs between the first and third quartiles, and the line inside the box represents the median; whiskers represent the lowest or highest values within 1.5 times IQR from the first or third quartiles. (B) Co-occurrence (Orange) and co-excluding (Green) relationships between bacterial species in Control and RNET groups. FastSpar correlation coefficients were presented by edge width (roh < -0.2 or roh > 0.2, *p* < 0.05). Nodes' size (Control: blue; RNET: dark red) were scaled based on the relative abundance of each microorganism in either RNET or Control group.

To further dissect the taxonomic profiling of individual's gut microbiome differentially abundant in RNET, MetaPhlAn2 annotation was run to assess the microbial abundance at the species level. In total, 641 microbial species representing 217 genera were profiled in 58 faecal samples (Figure S3), 24 of which differed significantly between RNET and control groups (Figure 2A). Notably, we observed a remarkable depletion of microbial species in RNET as compared with controls, which was consistent with the general trend towards loss of species diversity in several digestive diseases' microbiome 16. Bacterium such as *Haemophilus parainfluenzae*, *Veillonella unclassified*, and *Streptococcus salivarius* were significantly abundant in the healthy group. *Haemophilus parainfluenzae* and *Streptococcus salivarius* were commensal bacterium colonized in upper respiratory tract and gut, with the latter being a probiotic strain closely related with low occurrence of colorectal cancer 20, 21. In contrast, species, primarily composed of *Erysipelotrichaceae bacterium_6_1_45*, *Varibaculum cambriense* and *Methanobrevibacter smithii* exhibited the strongest enrichments in RNET subjects. *Varibaculum cambriense* is a medically related species involved in abscess formation 22, while overgrowth of *Methanobrevibacter smithii* was highly correlated with irritable bowel syndrome (IBS) 23-25. *Erysipelotrichaceae bacterium_6_1_45* belongs to the *Erysipelotrichaceae* family. Changes in the abundance level of such microorganism was observed in a spectrum of gastrointestinal diseases, including colorectal cancer, IBD, and TNF-driven Crohn's disease (CD) 26. Interestingly, previous studies strongly supported the close relation of these bacteria with metabolic disorders such as obesity. Highly abundant *Erysipelotrichaceae* was identified in obese individuals and in mice fed with high-fat diet 27, whereas germ-free mice colonized with *Methanobrevibacter smithii* showed enhanced degradation of dietary fructans, thereby promoting energy harvest and lipid storage 28. Linear discriminant analysis effect size (LEfSe) approach was further applied and identified 23 species that discriminately enriched in RNET or control groups (LEfSe: LDA > 2.0, *p* < 0.05). All these species were overlapped using the two methods (Figure S4A).

Microbial co-occurrence and co-excluding networks at the genus and species levels were constructed based on the Sparse Correlations using FastSpar 29. In contrast with healthy group, patients with RNET harbored a decreased complexity of network with lower connectivity. In the species network, we observed higher numbers of edge in control individuals (co-occurrence, 26; co-excluding, 17) compared with RNET patients (co-occurrence, 11; co-excluding, 5) (Figure 2B). In control subjects, RNET-enriched species, including *Erysipelotrichaceae bacterium_6_1_45*, *Varibaculum cambriense* and *Methanobrevibacter smithii*, showed the closest connectivity (roh > 0.6), which didn't recur in RNET group. *Haemophilus paraphrohaemolyticus* in healthy individuals contributed to most connection, including co-occurred with 4 species (*Neisseria subflava*, roh = 0.3106, *p* = 0.025; *Rothia aeria*, roh = 0.2768, *p* = 0.047; *Haemophilus sputorum*, roh = 0.3938, *p* = 0.01; *Actinobacillus unclassified*, roh = 0.5987, *p* = 0.001) and co-excluded with other 4 bacterium (*Atopobium parvulum*, roh = -0.3239, *p* = 0.02; *Granulicatella unclassified*, roh = -0.3506, *p* = 0.03; *Streptococcus salivarius*, roh = -0.3346, *p* = 0.024; *Veillonella unclassified*, roh = -0.3657, *p* = 0.024), highlighting its importance as a network-hub in control participants. By contrast, species network showed a less complexity in patients with RNET, implying the attenuated connection among these microorganisms, which may be due to the depleted microbial species in this group. Similar findings were found in the genus network (Figure S4B). We observed relatively higher connection among genera in control group as compared with RNET individuals.

### Functional characterization of the RNET microbiome

**Figure 3:**
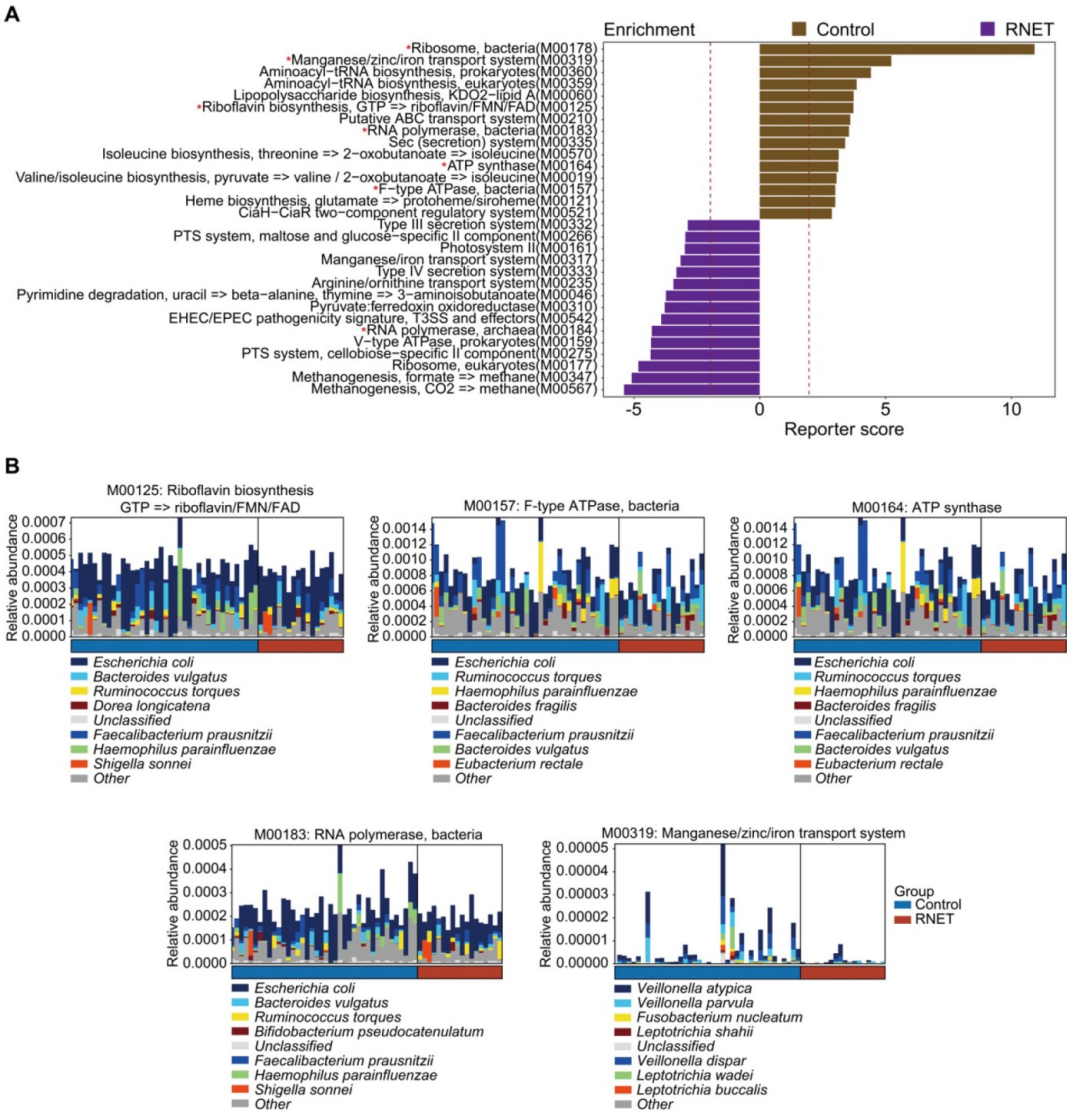
** Functional characterization of the RNET microbiome.** (A) The top 15 Kyoto Encyclopedia of Genes and Genomes (KEGG) modules annotated by our *in-house* pipeline were differentially abundant either in control or RNET groups (LDA > 2.0, *p* < 0.05). Modules overlapped with those annotated by the HUMAnN2 pipeline were marked with red asterisk (*). (B) Dominant taxonomic contributors for 5 KEGG modules simultaneously enriched by our *in-house* and HUMAnN2 pipelines, including M00125, M00157, M00164, M00183 and M00319 were predicted using the HUMAnN2 analysis. Species are proportionally scaled within the total bar height.

Next, we characterized the species-level functional outcomes of microbial changes in RNET through annotation of KEGG modules based on our *in-house* and the Human Project Unified Metabolic Analysis Network 2 (HUMAnN2) pipelines 30, aiming at providing a more comprehensive and accurate profiling of the molecular activities of microbial communities at the pathway perspective. In total, 69 KEGG modules (based on our *in-house* pipeline) and 38 KEGG modules (based on the HUMAnN2 pipeline) were differentially abundant between RNET and control groups (LEfSe: *p* < 0.05, LDA > 2.0), 11 of which overlapped using these two methods (Figure 3A, Figure S5). Probably due to the depleted microbial community, we only identified 3 KEGG modules enriched in RNET samples based on the HUMAnN2 pipeline, including M00378 (F420 biosynthesis), M00184 (RNA polymerase, archaea), and M00145 (NAD(P)H:quinone oxidoreductase, chloroplasts and cyanobacteria), resulting in the overlapped pathways mostly enriched from healthy individuals. Within the 11 pathways highlighted in both pipelines, those accounting for energy metabolism (M00157, M00164), RNA polymerase (M00183) and vitamin biosynthesis (M00125) were particularly enriched in control participants. We further identified species contributing predominantly to the highlighted pathways based on the output of HUMAnN2. Resultingly, we found that, despite the dominant species between these two groups were identical, their proportions were different (Figure 3B). A cluster of microorganisms mostly abundant in healthy people, including *Escherichia coli*, *Faecalibacterium prausnitzii*, *Bacteroides vulgatus*, *Haemophilus parainfluenzae*, *Ruminococcus torques* were the leading contributors to the above pathways. In contrast, Manganese/zinc/iron transportation (M00319) also enriched in control individuals was mainly contributed by species belonging to *Veillonella* genera, including *Veillonella atypica*, *Veillonella dispar* and *Veillonella parvula*. Overall, our findings indicate that shifts in microbial community composition drove a disease-liked state through interference with physiological functions.

### Metabolite enrichments in patients with RNET

**Figure 4:**
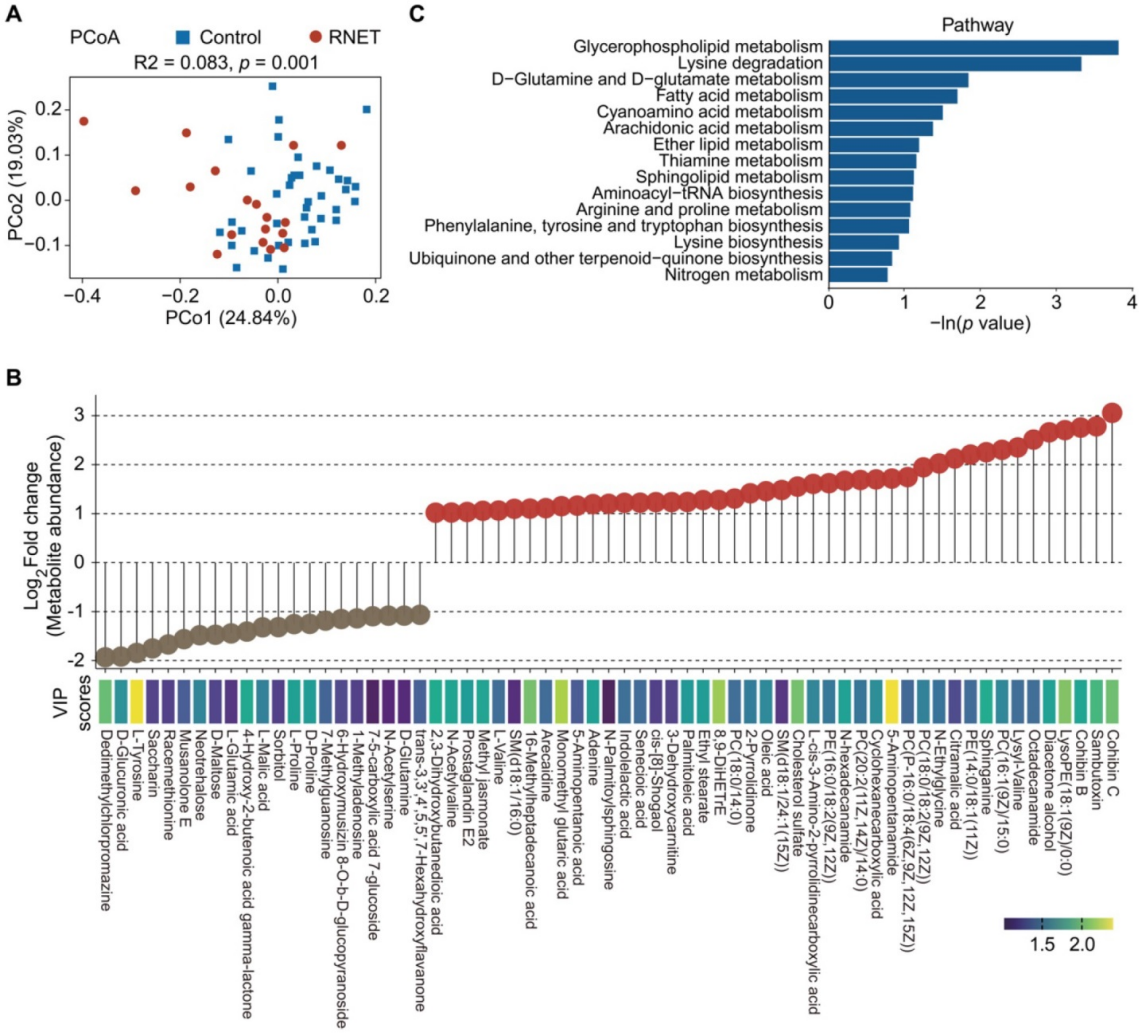
** Metabolite enrichments in patients with RNET.** (A) PCoA with Bray-Curtis distance in RNET and control groups based on faecal metabolic profiles. *p* values were calculated by two-sided Wilcoxon rank sum test. ADONIS, R^2^ = 0.083, *p* = 0.001. (B) Faecal metabolites (Log_2_ FC >1 or < -1, VIP > 1.0, *p* < 0.05, two-sided Wilcoxon rank-sum test) differentially abundant in RNET or healthy individuals were presented as lollipop chart. The length of line indicated the relative abundance of metabolites detected by LC-MS metabolomics. VIP score was indicated by a color gradient from purple (small numerical value) to yellow (large numerical value). (C) Bar plots of the top 15 KEGG pathways based on the In (*p* value) that biologically enriched from RNET-specific metabolites as compared with control subjects.

To further explore metabolic changes in RNET, untargeted LC-MS metabolomics was applied to detect metabolomes profiles of stool samples from RNET and healthy individuals. PCoA analysis displayed a clear separation of metabolite features between these two groups (adonis: R^2^ = 0.083, *p* = 0.001, Figure 4A). After adjusting for gender, BMI, age, smoking and alcohol consumption, we showed that Bray-Curtis similarities were not significantly affected by these parameters (Table S6). Similarly, we also observed significant dissociation by using the orthogonal projections to latent structures-discriminant analysis (OPLS-DA) to assess the quantitative variation in the metabolites between groups (anosim: R = 0.149, *p* = 0.026, Figure S6A), indicating broad metabolic difference between RNET and control samples. Enrichment analysis revealed a total of 545 faecal metabolites (Figure S7), 104 of which showing significantly different abundances between groups (*p* < 0.05, the projection value (VIP) > 1.0, Figure 4B). Contradicted to depleted microbial communities in RNET patients, most differential metabolites were remarkably abundant in stool samples from RNET patients (78 metabolites) versus control participants (26 metabolites). Differential metabolites were then assigned to putative molecular superclasses based on comparisons with the Human Metabolome Database (HMDB). Intriguingly, we found a total of 38 lipids and lipid-like molecules markedly up-regulated in RNET samples (Figure S6B). Among them, 57.9% (22 of 38) were fatty acyls such as Cohibin C, Cohibin B and Citramalic acid; 26.3% (10 of 38) were glycerophospholipids, including LysoPE (18:1(9Z)/0:0), PC (16:1(9Z)/15:0), and PE (14:0/18:1(11Z)) (Figure S6C). Cohibin C and Cohibin B, belonging to the annonaceous acetogenin group, were mitochondrial complex I inhibitors exerting a range of biological activities, including cytotoxic, immunosuppressive and antiparasitic properties 31, 32. By contrast, organoheterocyclic compounds, organic acids and derivates and organic nitrogen compounds showed distinctively up-regulated in control subjects. KEGG analysis further revealed that glycerophospholipid metabolism as key pathway was significantly altered in patients with RNET (Figure 4C). Together, our results suggested that dysregulation of lipid metabolism was involved in the pathogenesis of RNET.

### Putative correlation of gut microbial species with metabolites in RNET patients

**Figure 5:**
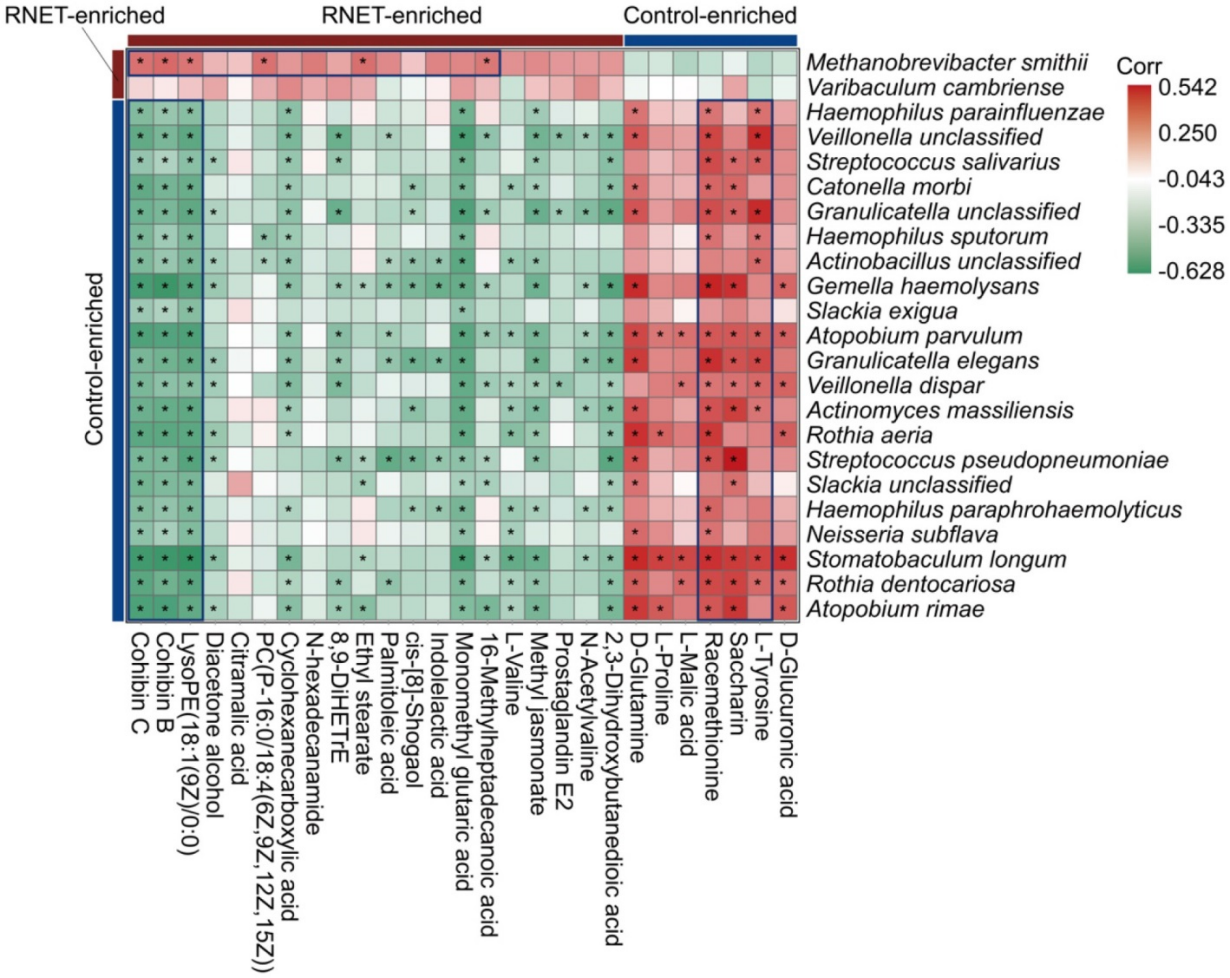
** Putative correlation of gut microbial species with metabolites in RNET patients.** Heatmap of Spearman's rank correlation coefficients between differentially abundant species (LEfSe: LDA > 2.0, *p* < 0.05) and metabolites (Log_2_ FC > 1 or < -1, VIP > 1.0, *p* < 0.05, *q* < 0.05) from RNET patients and controls. Correlation coefficients in each square represent positive (red) and negative (green) relationships. Statistically significant correlations (*p* < 0.05) were marked with asterisks (*). Representatively negative and positive correlations were labeled as deep blue squares.

Aberrations in gut metabolites have been tightly correlated with the pathophysiological processes contributing to IBD, obesity and colorectal cancer, which is a possible reverberation of perturbed microbiome community compositions and functions 33, 34. To understand the association of structure and metabolism of gut microbiota in RNET, we calculated the spearman's correlation of distinctively abundant species and metabolites, and presented a heatmap to highlight the species-metabolite-associated patterns (Figure 5). We revealed a total of 291 significant associations between representative differentially enriched species and metabolites. Roughly, positive correlations were detected between microorganisms and metabolites simultaneously enriched in RNET or controls, and vice versa. For example, *Methanobrevibacter smithii* was observed to be positively associated with several RNET-specific metabolites such as Cohibin B, Cohibin C and LysoPE(18:1(9Z)/0:0), corroborating the putative mechanistic relationship that positive correlations between species and metabolites could be explained by species producing metabolites, or metabolites benefiting the growth of certain species. Meanwhile, negative associations between control-enriched species and RNET-specific metabolites were also uncovered. Overall, although it remains to be further determined whether these metabolic products are directly metabolized by gut microbiota, our results demonstrate a tight interaction of gut microbial species with metabolites in RNET pathogenesis.

### Multi-omics signatures-based predication of RNET

**Figure 6:**
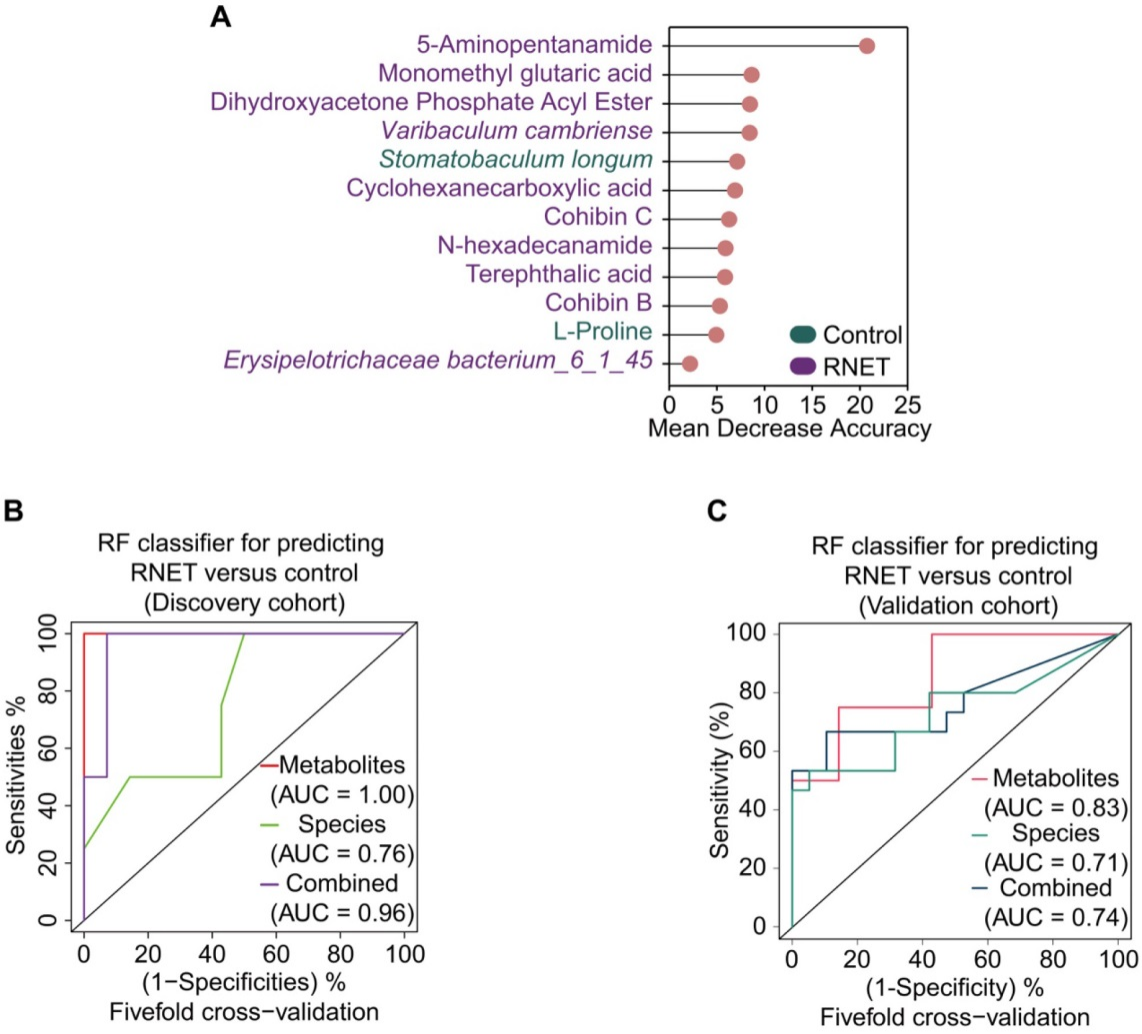
** Multi-omics signatures-based predication of RNET.** (A) 12 faecal microbial and metabolic biomarkers from RNET and control individuals reached the lowest classifier error were obtained by the mean decrease accuracy tool from the random forests (RFs) algorithm, and ranked by their contributions to classification accuracy after permutation. The color of each biomarker indicates its enrichment in RNET (purple) or control (green) participants. (B-C) Receiver operating characteristic (ROC) curve of the RF model using discriminatory signatures (3 species, 9 metabolites) in the 58 samples of discovery cohort (B) or 34 samples of validation cohort (C). RF method was used with train function of R's caret package. For training set, five-fold cross-validation was applied with trainControl function. To compute and visualize AUC from ROC outcome, the pROC package was utilized.

To excavate potential diagnostic microbial and metabolic signatures, we explored whether certain microbial species and metabolites could be utilized to predict RNET status. To this end, we constructed random forest (RF) classifiers based on the differential metabolic and microbial species profiles from control and RNET samples. Classification performance was evaluated using five-fold cross-validation and receiver operating characteristic curves. Notably, this approach enabled us to obtain a panel of gut microbiome and metabolite signatures composed of 3 species and 9 metabolic products, most of which were distinctively abundant in RNET patients (Figure 6A). The identified disease signatures included RNET-specific microbial species such as *Varibaculum cambriense* and *Erysipelotrichaceae bacterium_6_1_45*, and a cluster of metabolites. Among these, 5-Aminopentanamide, enriched in RNET, was the most discriminatory signature for the association with RNET. *Stomatobaculum longum* was the only taxonomic species significantly abundant in healthy group. Likewise, we adjusted these microbial and metabolic based classifiers for gender, age, BMI, smoking and alcohol consumption history, revealing that the identified biomarkers were not significantly influenced by these parameters (Table S7).

All RF classifiers performed markedly better than random in differentiating RNET and controls (Figure 6B). In particular, prediction model pointed out that metabolic signatures attained a high sensitivity in detecting RNET in the discovery cohort, with area under the curve (AUC) value of 1.0. Combination of microbial species and metabolites produced a remarkable improvement in classification accuracy compared with species alone (AUC = 0.96 versus 0.76). Next, we validated this discriminatory model on an additional independent cohort consisting of 15 RNET patient without receiving preoperative chemoradiotherapy and 19 healthy individuals (Figure 6C). Metabolic signatures still achieved higher accuracy for distinguishing RNET patients from controls with an AUC of 0.83. After combing these with microbial markers, the AUC value still reached to 0.74, which is better than microbiota biomarkers alone (AUC = 0.71). Thus, our results suggest the great potential for developing these microbial and metabolic based classifiers as a promising non-invasive tool in early fecal detection of populations with RNET.

## Discussion

US SEER database highlighted a substantially increased prevalence of RNET during the last decades, however, no advancement in outcome was traced over a similar period 35. One possible reason is the lack of messages about mechanistic alterations underlying the development of RNET. To date, apart from a handful of studies focusing on the genetic and epigenetic changes in such neoplasms 36, comprehensive mechanistic characterization is still urgently needed for therapeutic and diagnostic improvement for patients with RNET. To this end, we hereby presented a comprehensive and in-depth study by performing shotgun metagenomic and untargeted metabolomic profiling of faecal samples from RNET and healthy individuals. To the best of our knowledge, this study represents the first effort to elaborate RNET-specific alteration in human gut microbiome and metabolome in an integrated multi-omics framework.

In general, our study identified dysbiotic gut microbial and metabolic profiles in patients with RNET, although we didn't find a distinct shift of microbial community structure, as evidenced by multiple bacterial diversity analysis. However, aberrant depletion and attenuated connection of microbial species were uncovered in these RNET subjects, which was reminiscent of numerous publications indicating a similar loss of microbial diversity in a range of microbiota-associated diseases such as colorectal cancer 15, obesity 33 and IBD 16, 37, suggesting a pivotal role for the microbiota in health maintenance.

Gut microbiota are important for energy balance, for its influential behavior in enabling the host for energy harvest from diet 38. For example, Bäckhed *et al*. showed that fecal transplantation of gut microbiota from obese donors to germ-free mice led to rapid weight gain, due to the recovered capability of energy uptake 39. Our current study unveiled a nutrient deficient condition in gut microenvironment of RNET patients, which was supported by the metagenome-based functional analysis, showing diminished molecular activities such as energy metabolism, vitamin biosynthesis and transportation in RNET individuals. Such reduction was mostly attributed to the changed proportion of microbial species, but not maladjusted taxonomic composition. In particular, we discovered a weakened biosynthesis capacity of riboflavin (also known as vitamin B2) in patients with RNET. Higher vitamin B2 intake has been strongly associated with decreased risk for colorectal cancer and cardiovascular diseases 40-42, although its beneficial role in CRC was contradicted by a recent inspection 43. Future comprehensive investigations including the impact of preoperative and postoperative supplemental diet of this vitamin would help to validate its involvement in RNET pathogenesis.

Lipids have long since been recognized not just as critical substances of membrane structure and reservoir for energy storage, but also the key mediator to trigger diverse physiological processes 44. A large body of evidence coordinates the involvement of lipid molecules such as fatty acyls and glycerophospholipids in all phases of inflammation through interacting with cell surface or intracellular sensors to regulate inflammatory cell signaling and gene expression, therefore contributing to disease progression. Dysregulated lipid metabolism is now recognized a hallmark characteristic of many malignancies such as cardiovascular diseases, diabetes, obesity, and cancers 45-47. The lipid metabolic pathways that have been affected by diseases include synthesis, desaturation, elongation, and mitochondrial oxidation of lipid molecules 48. Our current faecal metabolomic analysis indicated a markedly aberrant accumulation of lipid and lipid-like compounds in RNET individuals, most of them could be assigned to the classes of fatty acyls and glycerophospholipids. KEGG analysis further implies glycerophospholipid metabolism as key pathway in RNET pathogenesis. Moreover, the correlation network exhibited a predominant association between gut microorganisms and metabolites. Thus, our findings proposed a possible implication of these differentially abundant intestinal flora and metabolic compounds in the onset and development of RNET, a process probably correlated with chronic inflammation. Although these putative association needs further *in vivo* and *in vitro* validation, our multi-omic results offered a guide for future exploration of causal relationship between key microbial species and metabolites in the development and progression of RNET.

Currently, we still lack of applicable, non-invasive approach for early diagnosis of RNET. Our present study identified a cluster of gut microbiome and metabolite-based signatures largely enriched in RNET individuals, showing accurate prediction of such neoplasm from healthy people, which was further validated in an independent cohort. Intriguingly, a core set of metabolic compounds mainly derived from RNET such as 5-Aminopentanamide achieved more reliable diagnostic accuracy relative to microbial signatures. Therefore, our findings may provide evidence for a stool-based diagnostic test for RNET among high-risk population.

Meanwhile, we acknowledge that limitations still exist in the current study. First, we included relatively small sizes of discovery and validation cohorts, mainly because of the small number of individuals affected by this neoplasm. Future multi-center studies comprising of geographically separated cohorts with well-phenotyped patients will be needed for further corroboration of these findings. Secondly, our findings are mostly data-driven, further *in vivo* and *in vitro* experiments are required for downstream validation. However, to date, the establishment and application of RNET disease models are still limited 49. In addition, although most RNET were detected as low-grade with well-differentiated histological morphology (Tumor grade of all RNET participants in our cohort were G1), patients with poorly-differentiated rectal neuroendocrine carcinomas need to be further recruited, for the purpose of better understanding the molecular pathogenesis of this disease.

In summary, our current study delineates a dysbiotic gut ecological microenvironment in patients with RNET, characterized by depleted microbial species and aberrantly aggregated lipids and lipid-like molecules. Although it remains to be further investigated whether these specific species and metabolic compounds directly cause tumorigenesis, disordered ecological structures may contribute to the oncogenic process of such neoplasm. Thus, our findings extend our insights to a potential role of disrupted gut microbiota and metabolites in the pathogenesis of RNET, which may provide promising targets for microbiome-based diagnostics and therapies.

## Supplementary Material

Supplementary materials and methods, figures.

Supplementary tables.

## Abbreviations

ANOSIM: Analysis of similarities
AUC: Area under the curve
BMI: Body mass index
CD: Crohn's disease
CgA: Chromogranin A
GEP-MENs: Gastroenteropancreatic neuroendocrine neoplasms
HMDB: Human Metabolome Database
HUMAnN2: Human Project Unified Metabolic Analysis Network 2
LC-MS: Liquid chromatography-mass spectrometry
LEfSe: Linear discriminant analysis effect size
IGC: Integrated Gene Catalog
MetaPhlAn2: Metagenomic phylogenetic analysis
NMDS: Nonmetric MultiDimensional Scaling
OPLS-DA: Orthogonal projections to latent structures-discriminant analysis
PCA: Principal Component Analysis
PCoA: Principal Coordinates analysis
SCFA: Short chain fatty acids
SEER: Surveillance, Epidemiology, and End Results
SYN: Synaptophysin
RF: Random forest
RNET: Rectal neuroendocrine tumors
VIP: The projection value
