# Supplementary material for: Faecal microbiome and metabolic signatures in rectal neuroendocrine tumors
Source: Theranostics. 2022 Jan 31;12(5):2015–27. doi: 10.7150/thno.66464 (PMC8899573; doi:10.7150/thno.66464)

Supplementary materials and methods

Study participants and faecal sample collection

All stool samples and clinical information utilized in this study were collected from patients at the Department of Gastroenterology, Shenzhen Hospital, Southern Medical University (Guangdong, China). Patient inclusion criteria include subjects aged > 18 with a diagnosis of RNET defined by endoscopy and histology examinations. None of these patients used antibiotics, proton pump inhibitors or probiotics at least 3 months before sample collection. Patients didn't receive preoperative chemotherapy or radiotherapy prior to the collection of samples. Healthy group comprise individuals undergoing colonoscopy for screening for polyp, colorectal cancer, or for physical examination at local hospitals, or any individuals who were interested to participate in this study. Participants who have hypertension, diabetes, liver diseases or long-term medication history were excluded in this study. Fresh samples were frozen in liquid nitrogen immediately and stored at -80 °C. All protocols were approved by the Ethic Committee of Southern Medical University (NYSZYYEC20190013) after obtaining patients' informed consent.

Stool sample DNA extraction

Stool sample DNA was extracted at Novogene Bioinformatics Technology (Beijing, China) using the SDS method. DNA was subsequently diluted to 1 ng/μl using sterile ddH_2O, and its degradation degree and contamination were assessed on 1% agarose gels. DNA purity (OD260/OD280) was determined using the NanoDrop Microvolume Spectrophotometer (Thermo Fisher Scientific, USA). DNA concentration was measured using the Qubit® dsDNA Assay Kit in Qubit® 2.0 Fluorometer (Life Technologies, Carlsbad, CA, USA).

Metagenomic shotgun sequencing

All samples were sequenced on an Illumina platform in PE150 mode at Novogene Bioinformatics Technology (Beijing, China). Adapter was trimmed and low-quality reads were filtered using trimmomatic-0.39. Then, host sequences were removed by aligning sequencing reads back to host genome reference (hg38) using soap2 (version 2.20) when sequence identity exceeds 90% [1].

Taxonomic profiling of the metagenomic samples was performed using MetaPhlAn2 [2] which uses clade-specific markers to provide pan-microbial (bacterial, archaeal, viral and eukaryotic) quantification at species-level. MetaPhlAn2 was run with default parameters.

At the same time, the high-quality reads were aligned to the updated gut microbiome gene catalog [3] using SOAP2 (version 2.20) with a threshold of more than 90% identity and 95% reads length. Gene abundance profile was calculated as previously described [3]. Next, the relative abundances of KEGG (Kyoto Encyclopedia of Genes and Genomes) orthologous (KOs) groups were summed up from the relative abundances of their respective genes to obtain functional profile.

Bioinformatic analysis

Alpha diversity was measured by observed counts and Shannon index at gene, phylum, genus and species level, respectively, with an in-house Perl script. Bray-Curtis distance was calculated using python module scipy (1.5.1). Principal component analysis (PCA) was analyzed using R package FactoMineR and factoextra. Principal coordinates analysis (PCoA) was used for visualizing beta diversity using the Bray-Curtis distance matrix data in R with ggplot2. R packages vegan and ggplot2 was used to analyze and visualize NMDS using Bray-Curtis distance. PERMANOVA was also analyzed using R package vegan with permutation times of 999. LEfSe (Linear discriminant analysis Effect Size) was performed with LEfSe (version 1.0) software to determine the features most likely to explain differences between groups.

Random forest model was built to find biomarkers most likely to be related to BCS status with R package randomForest and pROC was used to perform ROC analysis on random forest models.

Differentially enriched KEGG pathways/modules were identified according to their reporter score from the Z-scores of individual KOs as previously described [4, 5]. Briefly, a one-tail Wilcoxon rank-sum test was performed on all the KOs that occurred in more than five samples and adjusted for multiple testing using the Benjamin-Hochberg procedure. The Z-score for each KO was then calculated. Z-score of pathway/module background distribution was corrected and used as the final report score for evaluating the enrichment status. A report score of ≥ 1.96 (95% confidence according to normal distribution) could be used as a detection threshold for significantly differentiating pathways.

HUMAnN2 analysis

Functional profiling was performed by HUMAnN2 [6]. Sample reads are mapped against this database to quantify gene presence and abundance on a per-species basis. A translated search is then performed against a UniRef-based protein sequence catalogue for all reads that fail to map at the nucleotide level. The result are abundance profiles of gene families (UniRef90s), stratified by each species contributing those genes, and which can then be summarized to higher-level gene groupings such as ECs or KOs.

Metabolites Extraction

20 mg of sample was weighted to an EP tube, and 1000 μL extract solution (methanol:acetonitrile:water = 2:2:1, with isotopically-labelled internal standard mixture) was added. Then the samples were homogenized at 35 Hz for 4 min and sonicated for 5 min in ice-water bath. The homogenization and sonication cycle were

repeated for 3 times. Then the samples were incubated for 1 hr at -40 °C and centrifuged at 12000 rpm for 15 min at 4 °C. The resulting supernatant was transferred to a fresh glass vial for analysis. The quality control (QC) sample was prepared by mixing an equal aliquot of the supernatants from all samples.

LC-MS/MS Analysis

LC-MS/MS analyses were performed using an UHPLC system (Vanquish, Thermo Fisher Scientific) with a UPLC BEH Amide column (2.1 mm × 100 mm, 1.7 μm) coupled to Q Exactive HFX mass spectrometer (Orbitrap MS, Thermo). The mobile phase consisted of 25 mmol/L ammonium acetate and 25 ammonia hydroxide in water (pH = 9.75) (A) and acetonitrile (B). The auto-sampler temperature was 4 °C, and the injection volume was 3 μL.

The QE HFX mass spectrometer was used for its ability to acquire MS/MS spectra on information-dependent acquisition (IDA) mode in the control of the acquisition software (Xcalibur, Thermo). In this mode, the acquisition software continuously evaluates the full scan MS spectrum. The ESI source conditions were set as following: sheath gas flow rate as 30 Arb, Aux gas flow rate as 25 Arb, capillary temperature 350 °C, full MS resolution as 60000, MS/MS resolution as 7500, collision energy as 10/30/60 in NCE mode, spray Voltage as 3.6 kV (positive) or -3.2 kV (negative), respectively.

Data preprocessing and annotation

The raw data were converted to the mzXML format using ProteoWizard and processed with an in-house program, which was developed using R and based on XCMS, for peak detection, extraction, alignment, and integration. Then an in-house MS2 database (BiotreeDB) was applied in metabolite annotation. The cutoff for annotation was set at 0.3.

Correlation between genus and species in each group by FastSpar

Microbial association (genus and species level) in each group was determined by FastSpar, a fast and parallelizable implementation of the SparCC algorithm with an unbiased P-value estimator [7]. We selected the significantly different genus and species between RNET and control groups ($p < 0.05$). FastSpar has been widely used to estimate the correlation values from compositional data. Significant co-occurrence and co-excluding interaction (FastSpar correlation scores roh < −0.2 or roh > 0.2, $p < 0.05$) were visualized and analyzed using igraph. We calculated the degree, betweenness and strength of each node to estimate its importance to the network.

Correlation analysis of gut microbial species and metabolites

We selected gut microbial species discriminately enriched in RNET or control groups by LEfSe analysis (LEfSe: LDA > 2.0, $p < 0.05$). Significantly abundant metabolites were defined as $\log_2$ Fold change (FC) > 1 or < -1, $p < 0.05$, $q < 0.05$. Consequently, 23 metabolites were included. Spearman's correlation of differentially enriched species and metabolites was calculated using the scipy-stats package. Heat maps were hierarchically clustered to represent the species-metabolite-associated patterns based on the correlation distance. All analyses and visualizations were implemented in python (v2.7.9) with the numpy (v1.9.2), scipy (v0.15.1), and matplotlib (v1.4.3) packages.

Statistical analysis

We performed Wilcoxon rank-sum test (two-tailed) for the difference of α diversity and permutation multivariate analysis of variance (PERMANOVA) test for the difference of β diversity between the two compared groups. Spearman correlation analysis between differential enriched features were performed. To evaluate and

deconfound the effects of gender, age, BMI, smoking and alcohol consumption, multivariate association with linear models algorithm (MaAsLin2, http://huttenhower.sph.harvard.edu/maaslin) was used for multivariable association testing between phenotypes and microbial taxonomy or functional characters with default parameters. Unless otherwise stated, all statistical analyses were made in the R software and P values < 0.05 were considered as statistically significant level.

Reference

1 Qin J, Li Y, Cai Z, Li S, Zhu J, Zhang F, et al. A metagenome-wide association study of gut microbiota in type 2 diabetes. Nature 2012;490:55-60.

2 Truong DT, Franzosa EA, Tickle TL, Scholz M, Weingart G, Pasolli E, et al. MetaPhlAn2 for enhanced metagenomic taxonomic profiling. Nat Methods 2015;12:902-3.

3 Li J, Jia H, Cai X, Zhong H, Feng Q, Sunagawa S, et al. An integrated catalog of reference genes in the human gut microbiome. Nat Biotechnol 2014;32:834-41.

4 Patil KR, Nielsen J. Uncovering transcriptional regulation of metabolism by using metabolic network topology. Proc Natl Acad Sci U S A 2005;102:2685-9.

5 Feng Q, Liang S, Jia H, Stadlmayr A, Tang L, Lan Z, et al. Gut microbiome development along the colorectal adenoma-carcinoma sequence. Nat Commun 2015;6:6528.

6 Franzosa EA, McIver LJ, Rahnavard G, Thompson LR, Schirmer M, Weingart G, et al. Species-level functional profiling of metagenomes and metatranscriptomes. Nat Methods 2018;15:962-8.

7 Watts SC, Ritchie SC, Inouye M, Holt KE. FastSpar: rapid and scalable correlation estimation for compositional data. Bioinformatics 2019;35:1064-6.

Supplementary Figure legends

Figure S1 (A) Representative images for 18 RNET patients (Colonoscopy and H&E histological pictures) and 40 healthy individuals (Colonoscopy pictures) in the discovery cohort were presented. (B) Distribution of BMI, age, Brinkman index (smoking) and alcohol consumption in 58 subjects (40 healthy individuals, 18 RNET) were calculated. The dot represents one value from individual participants. Lines in the boxes indicate medians, the width of the notches is the IQR, the lowest and highest values within 1.5 times the IQR from the first and third quartiles. *p* values were calculated by two-sided Wilcoxon rank sum test.

Figure S2 (A-C) Gut microbial structures in RNET patients and healthy participants were evaluated by Principal Component Analysis (PCA), Principal Coordinates analysis (PCoA) and Nonmetric MultiDimensional Scaling (NMDS) at the phylum (A), genus (B), and species (C) levels, respectively, based on the metagenomic data. (D) ANOSIM test was applied to compare microbial structure dissimilarity between and within groups (two-sided wilcoxon rank-sum test).

Figure S3 641 microbial species in RNET and control groups detected by metagenomic sequencing were normalized, centered, clustered, and presented by heatmap.

Figure S4 (A) Enriched species either in RNET or control group were presented by LEfSe bar plots, which were interpreted by linear discriminant analysis (LDA) scores (Logarithmic LDA score > 2.0, $p < 0.05$ were considered a significant difference in bacterial abundance between groups). (B) Co-occurrence (Orange) and co-excluding (Green) relationships between bacterial genera in Control and RNET groups. FastSpar correlation coefficients were indicated by edge width (roh < −0.2 or roh > 0.2, $p <$ 0.05). Nodes' size (Control: blue; RNET: dark red) were scaled based on the relative

abundance of each genus in either RNET or Control group.

Figure S5 The top 27 KEGG modules differentially abundant in control group and 3 KEGG modules enriched in RNET patients were annotated by the HUMAnN2 pipeline (p < 0.05 was considered as statistical significance, two-sided wilcoxon rank-sum test). Modules overlapped with those annotated by our *in-house* pipeline were marked with red asterisk (*).

Figure S6 (A) The orthogonal projections to latent structures-discriminant analysis (OPLS-DA) were applied to assess the quantitative variation in the metabolites between RNET and control groups. p values were calculated by two-sided Wilcoxon rank sum test. ANOSIM, R = 0.149, p = 0.026. (B) Metabolic compounds were assigned to putative molecular superclasses based on comparisons with the Human Metabolome Database (HMDB). Relative abundance of faecal molecular superclasses showed significant difference between RNET patients and healthy individuals. N represents the number of metabolites in each superclass (two-sided wilcoxon rank-sum test). (C) The number of class members in lipid and lipid-like molecules were presented.

Figure S7 545 faecal metabolites in RNET and control groups detected by metabonomic profiling were normalized, centered, clustered, and presented by heatmap.

Table S1 Participants' clinical information in the discovery cohort.

Table S2 Bacterial β-diversity calculated by PERMANOVA with Bray-Curtis distance.

Table S3 IGC-based gene count analysis adjusted by clinical parameters.

Table S4 MetaPhlAn2-based α-diversity analysis adjusted by clinical parameters.

Table S5 Bacterial β-diversity adjusted by clinical parameters.

Table S6 Metabolic bray-Curtis similarities adjusted by clinical parameters.

Table S7 Microbial and metabolic based classifiers adjusted by clinical parameters.

Figure S1

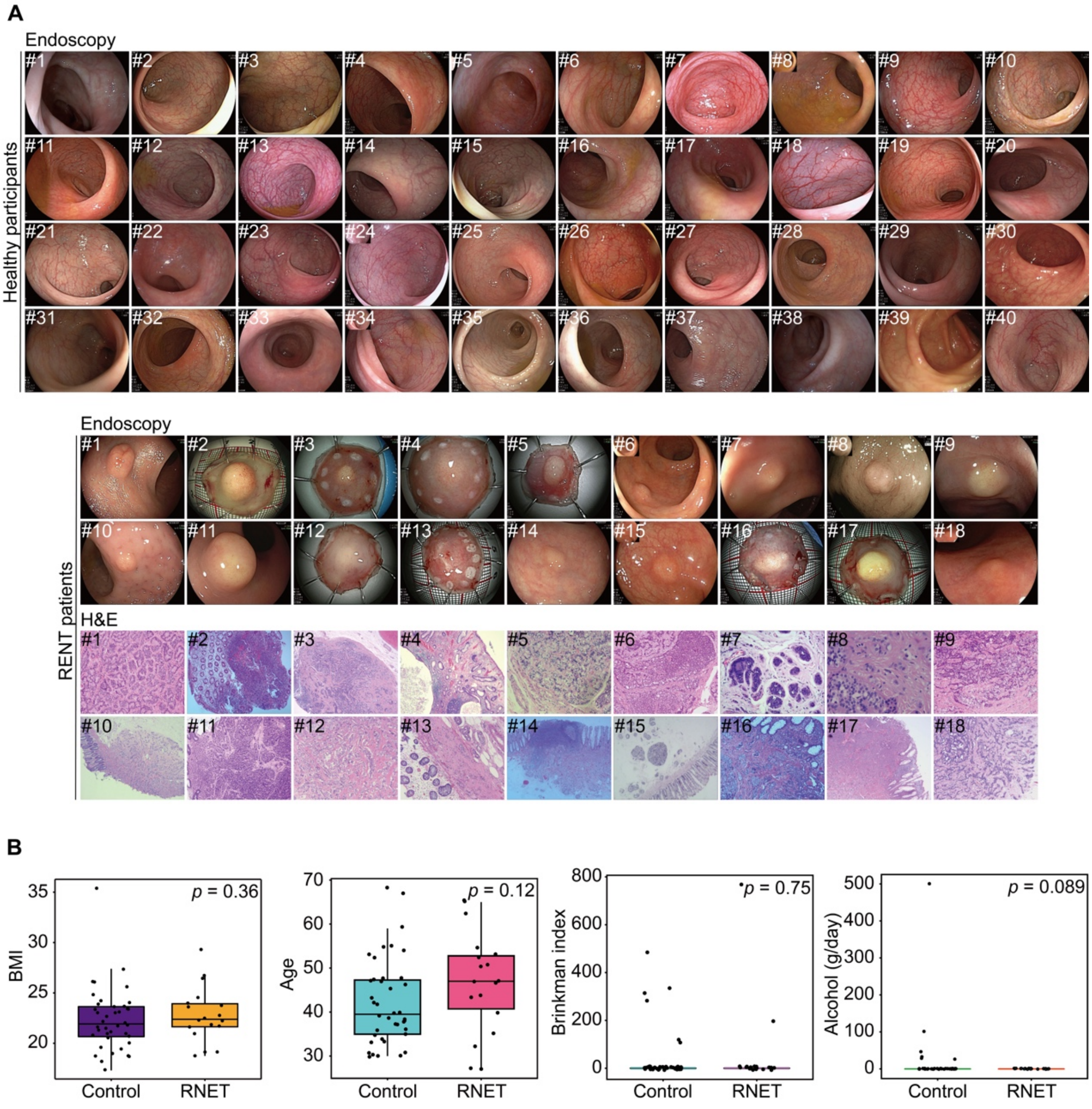
A
Endoscopy
Healthy participants
RENT patients
H&E
B
BMI
Age
Brinkman index
Alcohol (g/day)
Control
RNET
p = 0.36
p = 0.12
p = 0.75
p = 0.089

Figure S2

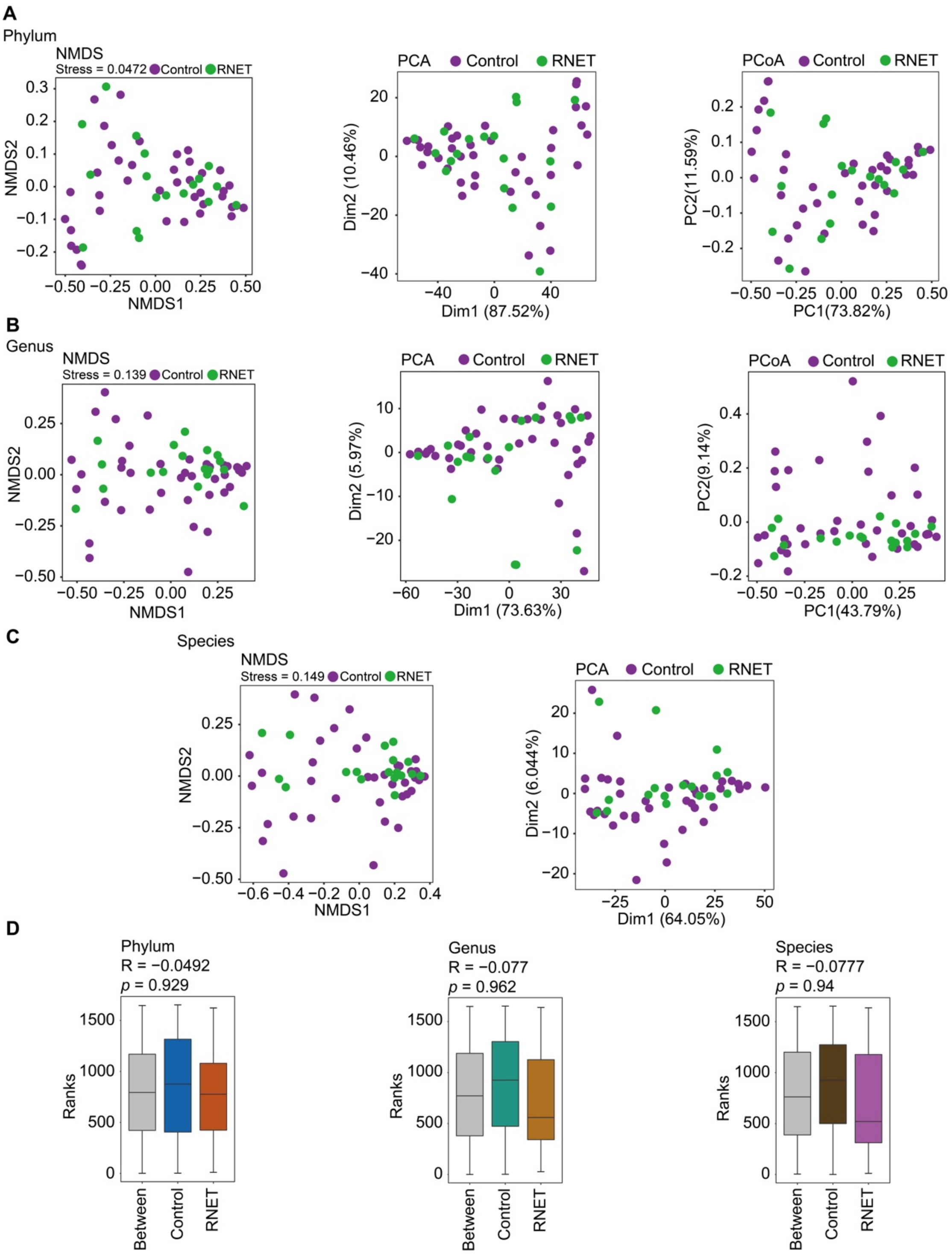

A
Phylum
NMDS
Stress = 0.0472 Control RNET
NMDS2
NMDS1
PCA Control RNET
Dim2 (10.46%)
Dim1 (87.52%)
PCoA Control RNET
PC2(11.59%)
PC1(73.82%)
B
Genus
NMDS
Stress = 0.139 Control RNET
NMDS2
NMDS1
PCA Control RNET
Dim2 (5.97%)
Dim1 (73.63%)
PCoA Control RNET
PC2(9.14%)
PC1(43.79%)
C
Species
NMDS
Stress = 0.149 Control RNET
NMDS2
NMDS1
PCA Control RNET
Dim2 (6.044%)
Dim1 (64.05%)
D
Phylum
R = −0.0492
p = 0.929
Genus
R = −0.077
p = 0.962
Species
R = −0.0777
p = 0.94
Ranks
Between
Control
RNET

Figure S3

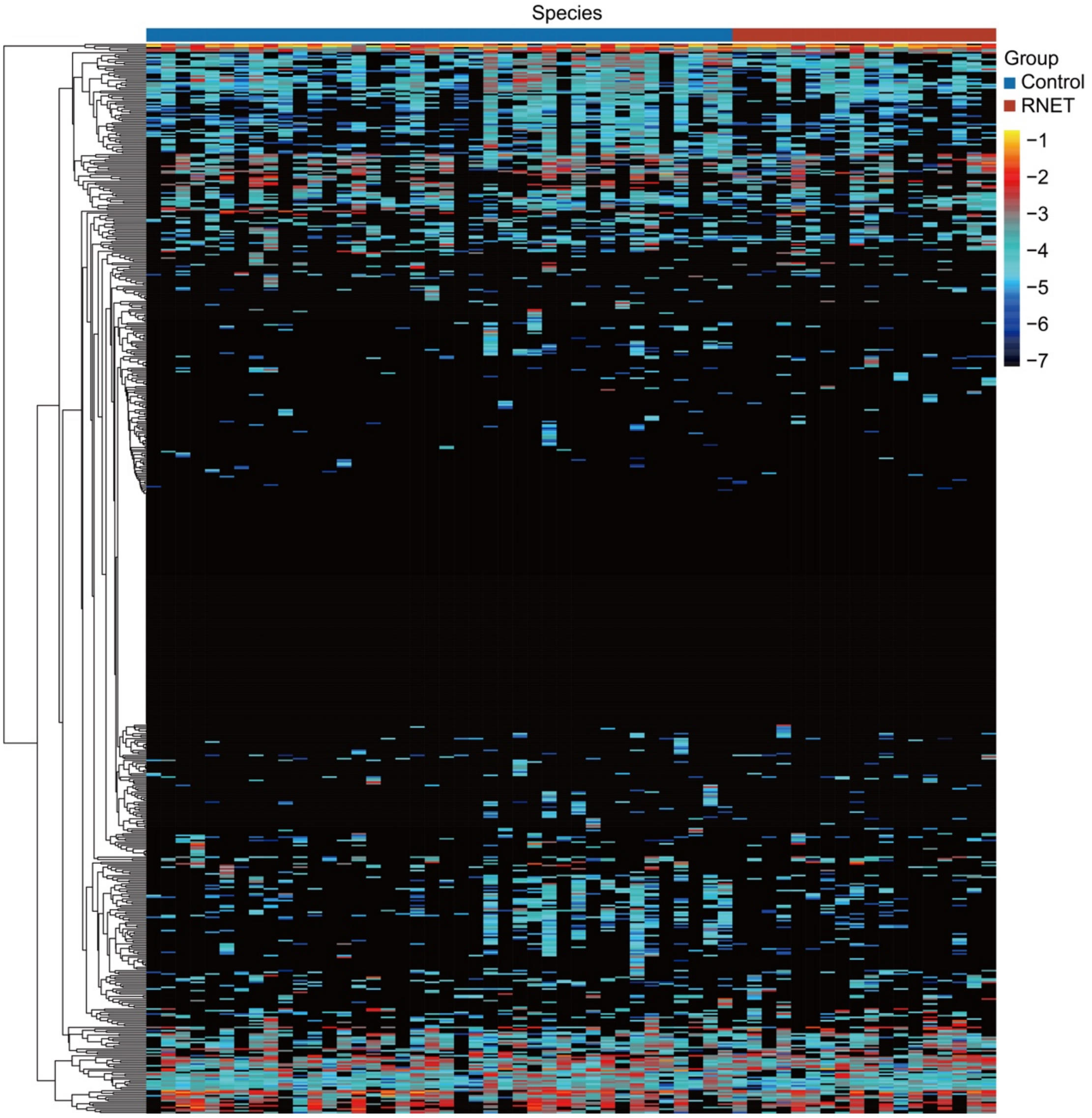

Figure S4

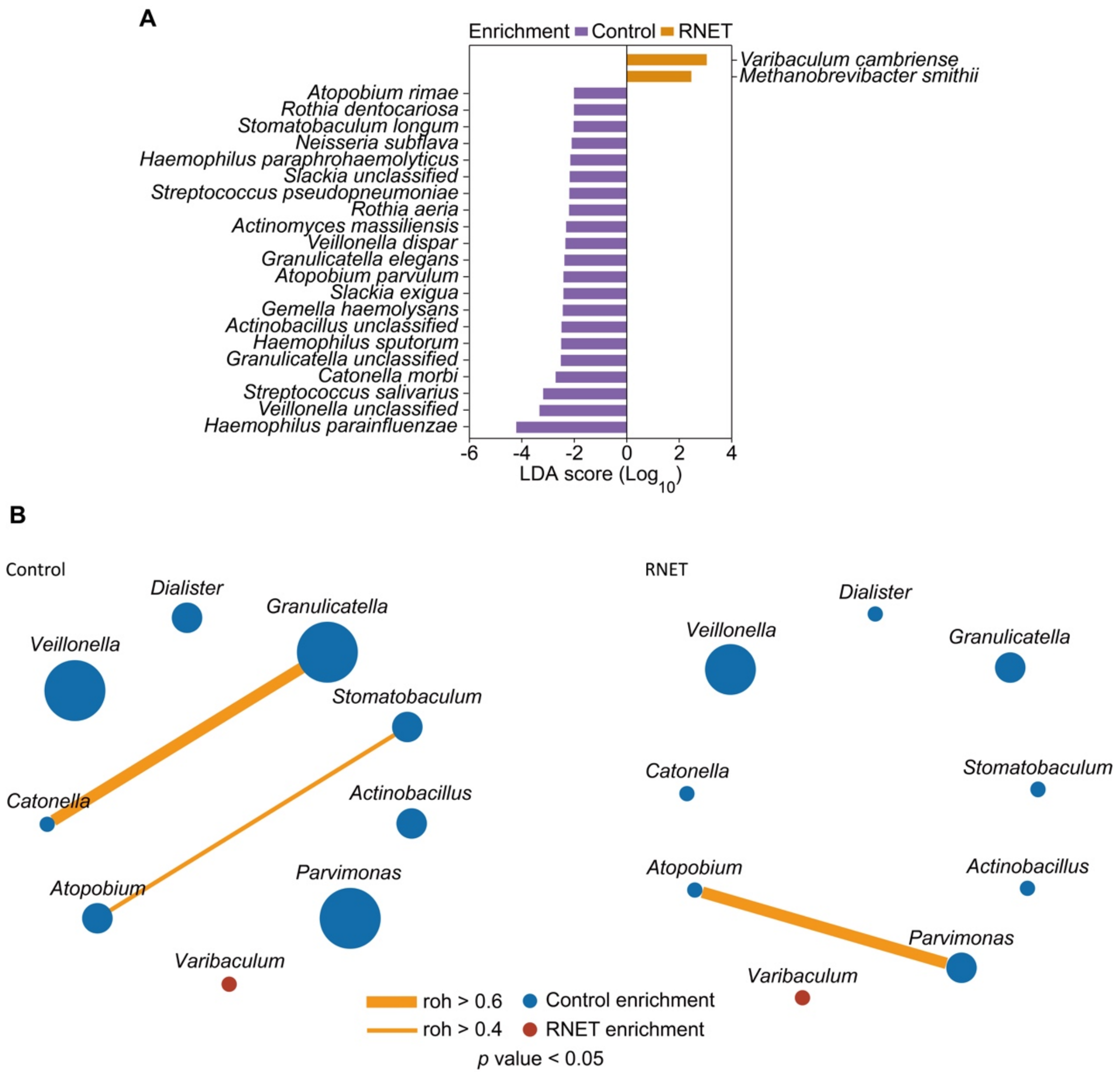
A
Enrichment Control RNET
Varibaculum cambriense
Methanobrevibacter smithii
Atopobium rimae
Rothia dentocariosa
Stomatobaculum longum
Neisseria subflava
Haemophilus paraphrohaemolyticus
Slackia unclassified
Streptococcus pseudopneumoniae
Rothia aeria
Actinomyces massiliensis
Veillonella dispar
Granulicatella elegans
Atopobium parvulum
Slackia exigua
Gemella haemolysans
Actinobacillus unclassified
Haemophilus sputorum
Granulicatella unclassified
Catonella morbi
Streptococcus salivarius
Veillonella unclassified
Haemophilus parainfluenzae
-6 -4 -2 0 2 4
LDA score (Log_{10})
B
Control
Dialister
Granulicatella
Veillonella
Stomatobaculum
Actinobacillus
Catonella
Parvimonas
Atopobium
Varibaculum
RNET
Dialister
Veillonella
Granulicatella
Catonella
Stomatobaculum
Atopobium
Actinobacillus
Parvimonas
Varibaculum
roh > 0.6
roh > 0.4
Control enrichment
RNET enrichment
p value < 0.05

Figure S5

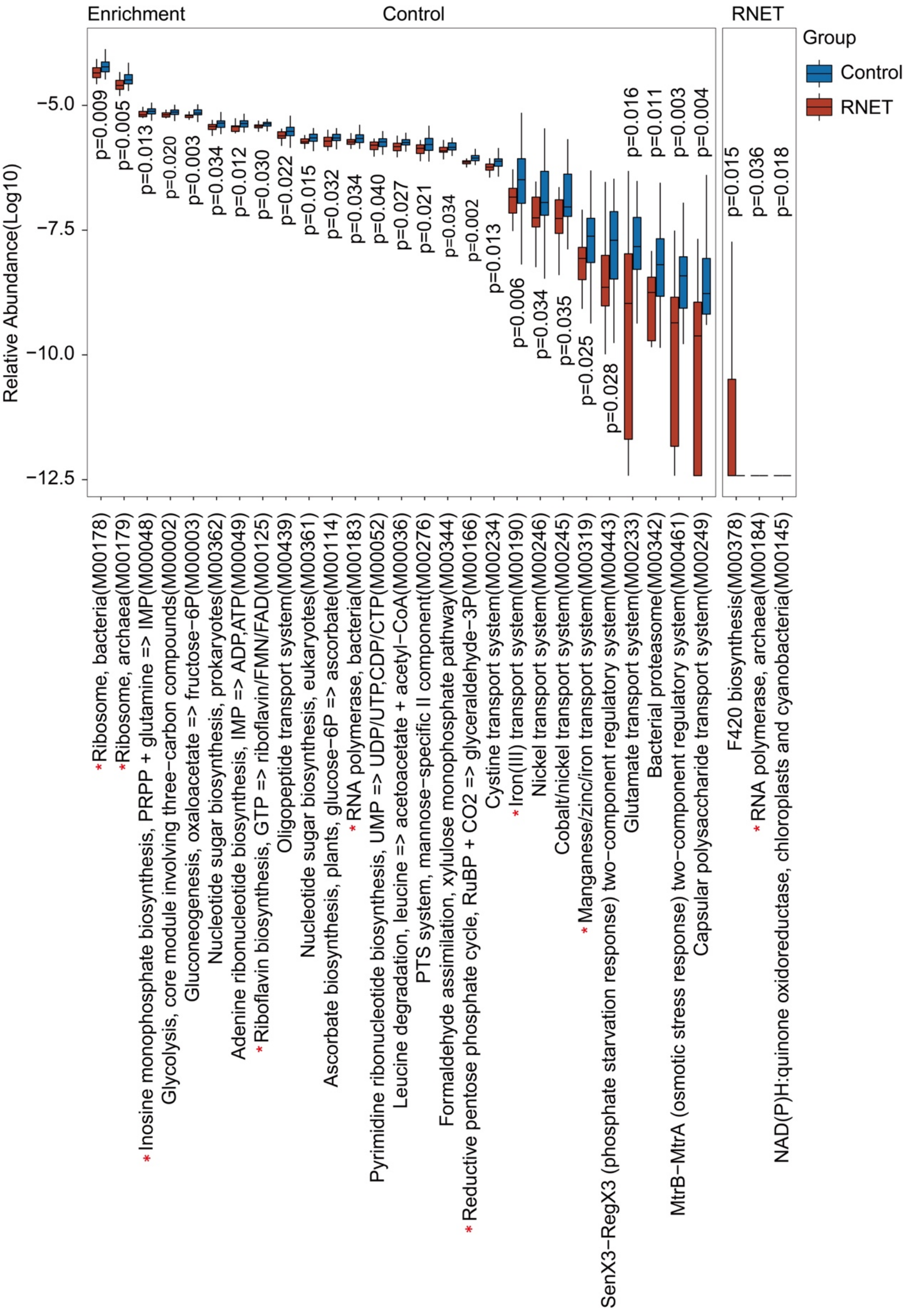

Enrichment
Control
RNET
Group
Control
RNET
Relative Abundance(Log10)
−5.0
−7.5
−10.0
−12.5
p=0.009
p=0.005
p=0.013
p=0.020
p=0.003
p=0.034
p=0.012
p=0.030
p=0.022
p=0.015
p=0.032
p=0.034
p=0.040
p=0.027
p=0.021
p=0.034
p=0.002
p=0.013
p=0.006
p=0.034
p=0.035
p=0.025
p=0.028
p=0.016
p=0.011
p=0.003
p=0.004
p=0.015
p=0.036
p=0.018
*Ribosome, bacteria(M00178)
*Ribosome, archaea(M00179)
*Inosine monophosphate biosynthesis, PRPP + glutamine => IMP(M00048)
Glycolysis, core module involving three−carbon compounds(M00002)
Gluconeogenesis, oxaloacetate => fructose−6P(M00003)
Nucleotide sugar biosynthesis, prokaryotes(M00362)
Adenine ribonucleotide biosynthesis, IMP => ADP,ATP(M00049)
*Riboflavin biosynthesis, GTP => riboflavin/FMN/FAD(M00125)
Oligopeptide transport system(M00439)
Nucleotide sugar biosynthesis, eukaryotes(M00361)
Ascorbate biosynthesis, plants, glucose−6P => ascorbate(M00114)
*RNA polymerase, bacteria(M00183)
Pyrimidine ribonucleotide biosynthesis, UMP => UDP/UTP,CDP/CTP(M00052)
Leucine degradation, leucine => acetoacetate + acetyl−CoA(M00036)
PTS system, mannose−specific II component(M00276)
Formaldehyde assimilation, xylulose monophosphate pathway(M00344)
*Reductive pentose phosphate cycle, RuBP + CO2 => glyceraldehyde−3P(M00166)
Cystine transport system(M00234)
*Iron(III) transport system(M00190)
Nickel transport system(M00246)
Cobalt/nickel transport system(M00245)
*Manganese/zinc/iron transport system(M00319)
SenX3−RegX3 (phosphate starvation response) two−component regulatory system(M00443)
Glutamate transport system(M00233)
Bacterial proteasome(M00342)
MtrB−MtrA (osmotic stress response) two−component regulatory system(M00461)
Capsular polysaccharide transport system(M00249)
F420 biosynthesis(M00378)
*RNA polymerase, archaea(M00184)
NAD(P)H:quinone oxidoreductase, chloroplasts and cyanobacteria(M00145)

Figure S6

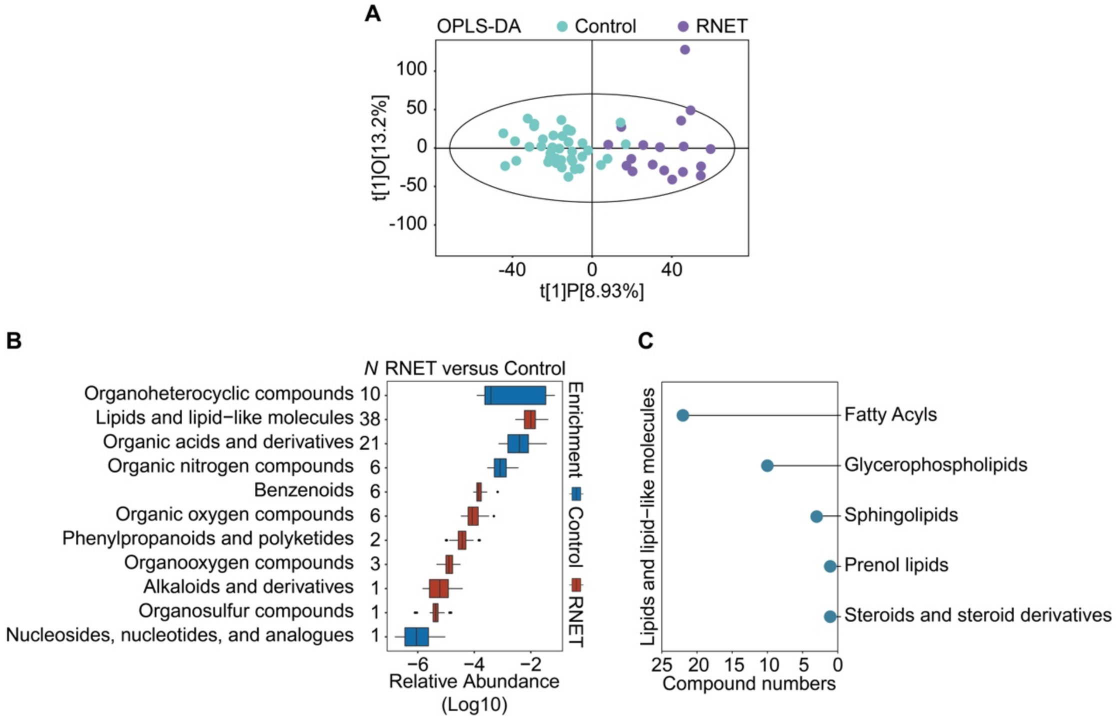
A
OPLS-DA
Control
RNET
t[1]O[13.2%]
t[1]P[8.93%]
100
50
0
-50
-100
-40
0
40
B
N RNET versus Control
Organoheterocyclic compounds 10
Lipids and lipid−like molecules 38
Organic acids and derivatives 21
Organic nitrogen compounds 6
Benzenoids 6
Organic oxygen compounds 6
Phenylpropanoids and polyketides 2
Organooxygen compounds 3
Alkaloids and derivatives 1
Organosulfur compounds 1
Nucleosides, nucleotides, and analogues 1
Enrichment
Control
RNET
−6
−4
−2
Relative Abundance
(Log10)
C
Lipids and lipid−like molecules
Fatty Acyls
Glycerophospholipids
Sphingolipids
Prenol lipids
Steroids and steroid derivatives
25
20
15
10
5
0
Compound numbers

Figure S7

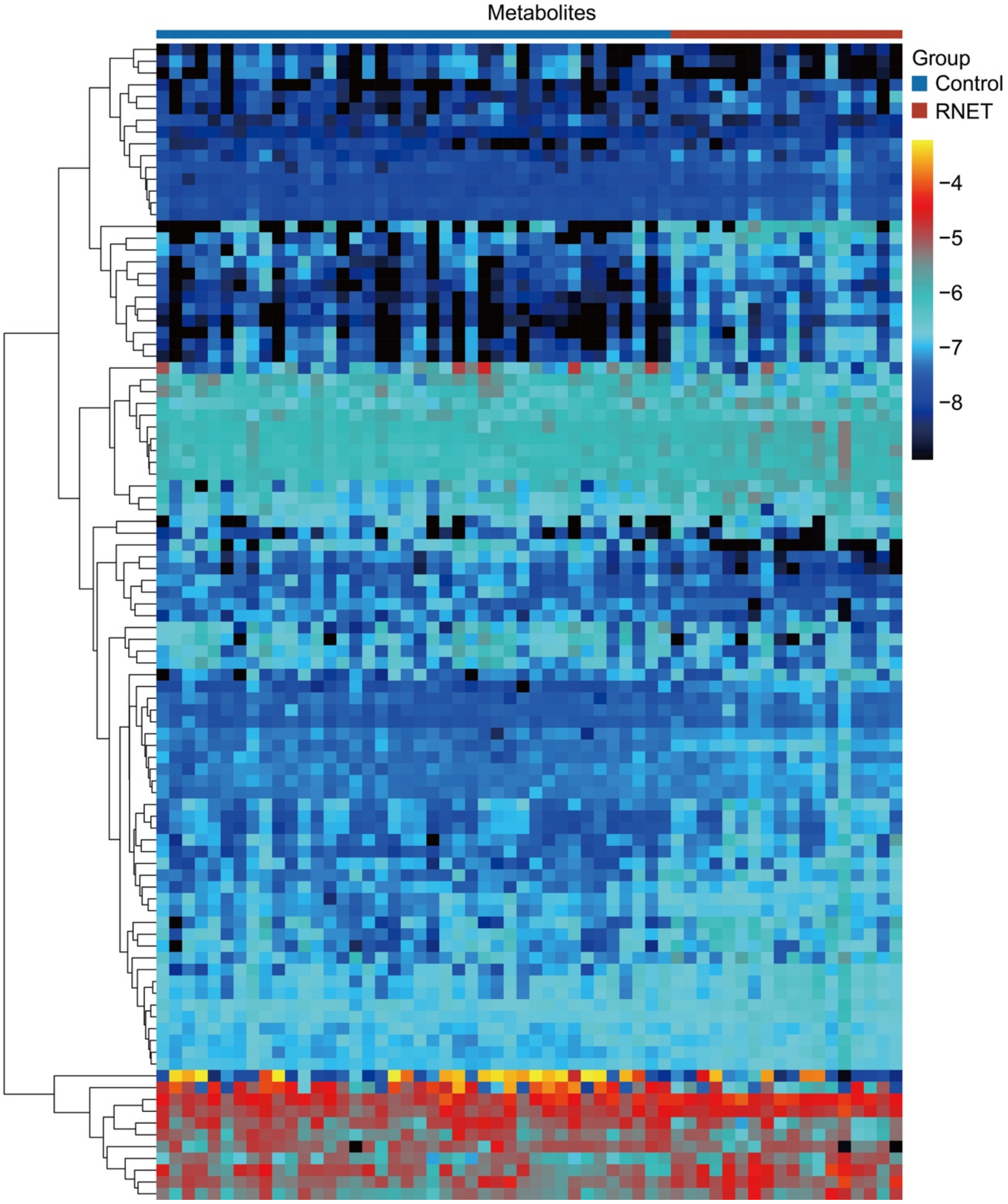

Supplement: Supplementary file 1 — Supplementary materials and methods, figures. [file thnov12p2015s1.pdf]
